# Supplementary material for: An extended interaction site determines binding between AP180 and AP2 in clathrin mediated endocytosis
Source: Nat Commun. 2024 Jul 13;15:5884. doi: 10.1038/s41467-024-50212-4 (PMC11246429; doi:10.1038/s41467-024-50212-4)
Supplement: Supplementary file 1 — Supplementary Information [file 41467_2024_50212_MOESM1_ESM.pdf]

Supplementary Information

An extended interaction site in the neuronal AP180 is responsible for recruitment of AP2 in clathrin mediated endocytosis

Samuel Naudi-Fabra *et al.*

*Corresponding author. Email: milles@fmp-berlin.de

Supplementary Figures:

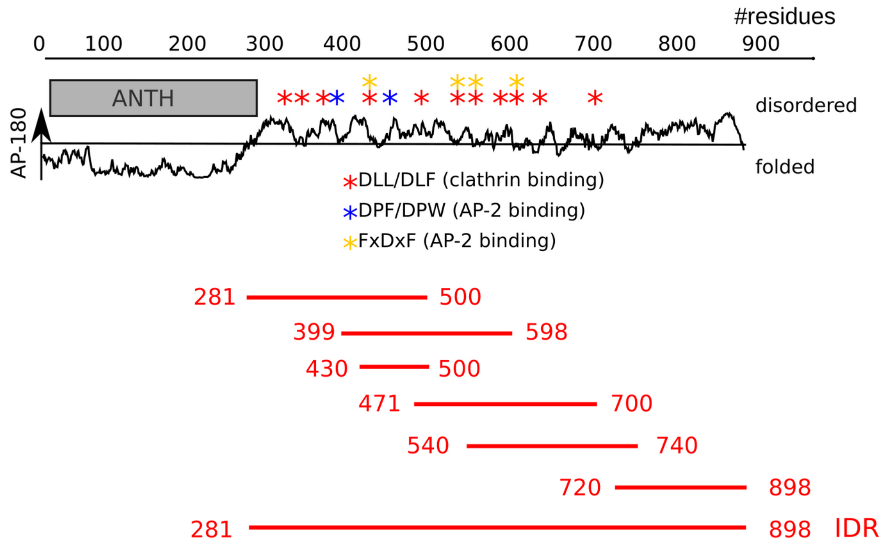

Supplementary Figure 1: Disorder analysis of AP180 and divide and conquer approach. Top: Disorder prediction (IUPred[1]) of AP180 along its sequence. The gray box represents the folded ANTH (AP180 N-terminal homology) domain. Stars above the disorder prediction represent putative interaction sites with the clathrin heavy chain terminal domain and with the AP2 appendage domains as indicated in the figure. Illustrated below are the constructs used in this study. The numbers on the left and on the right represent the N-terminal and C-terminal limits of the different constructs (called ‘segments’), respectively.

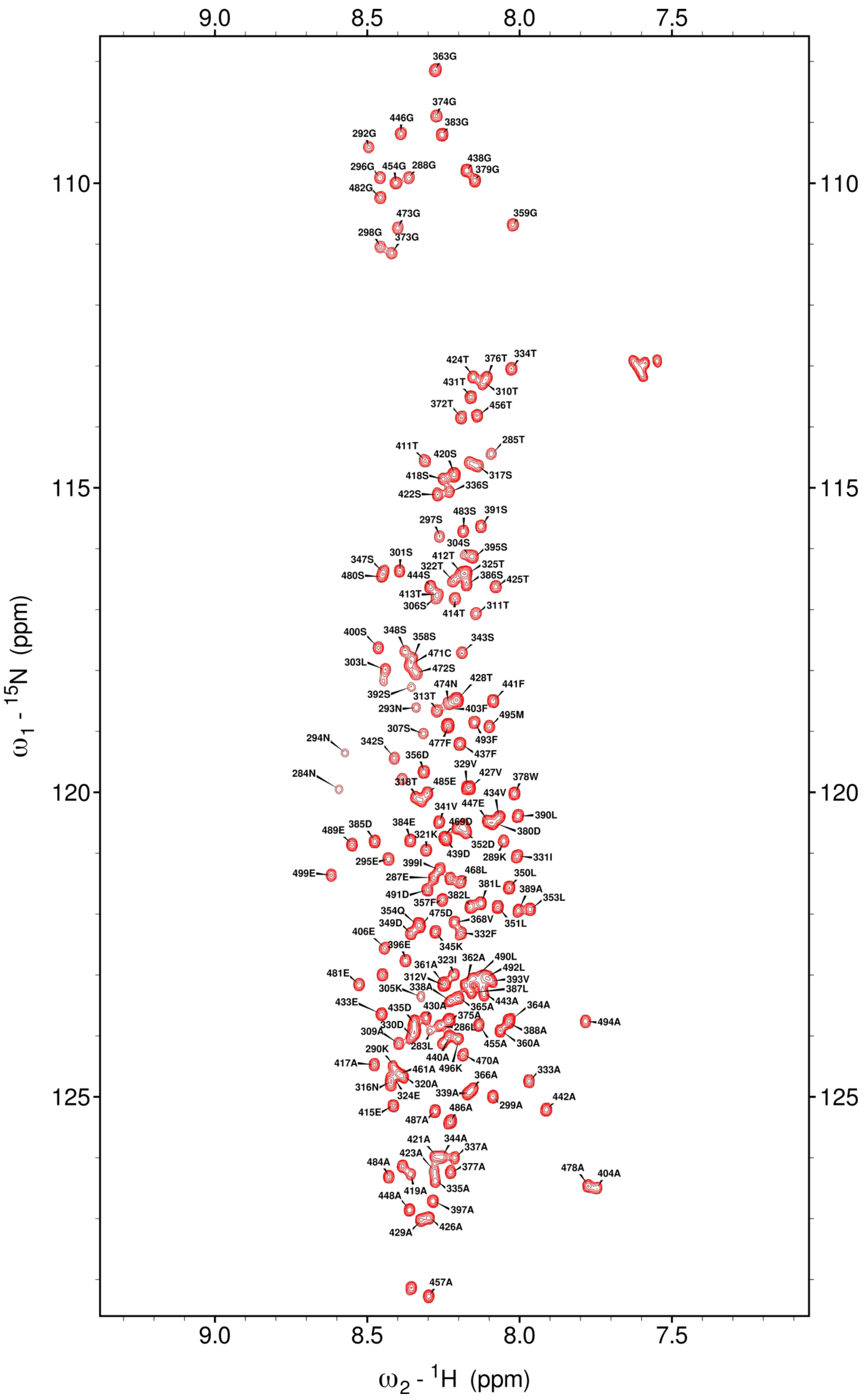

Supplementary Figure 2: Assignment of AP180$_{281\text{-}500}$. The ^{1}H-^{15}N HSQC of AP180$_{281\text{-}500}$ showing the peak assignment. Note that all numbers are shifted by +1 with respect to the protein sequence for cloning reasons. 83% of the non-proline residues have been assigned.

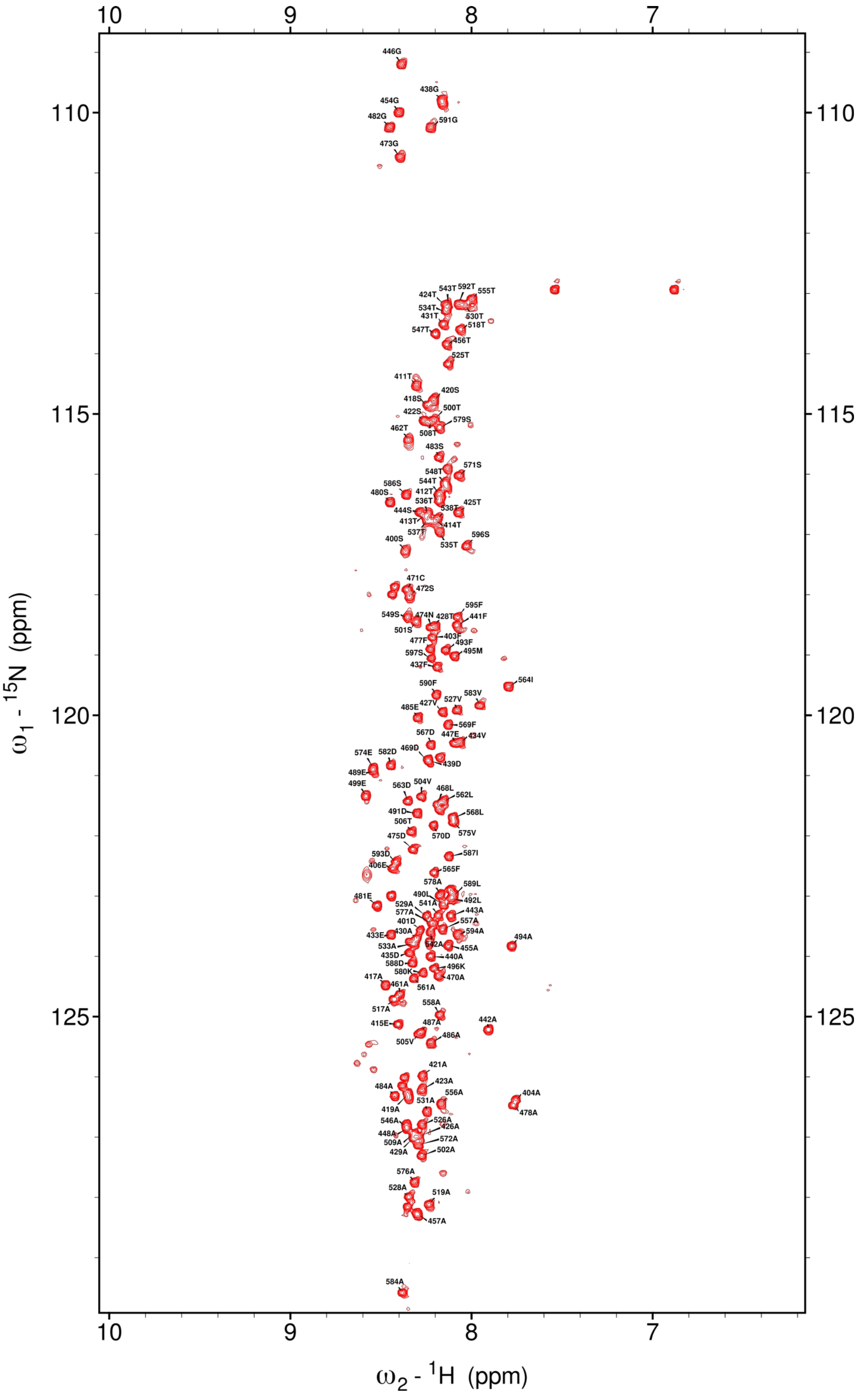

Supplementary Figure 3: Assignment of AP180$_{399\text{-}598}$. The ^{1}H-^{15}N HSQC of AP180$_{399\text{-}598}$ showing the peak assignment. Note that all numbers are shifted by +1 with respect to the protein sequence for cloning reasons. 81% of the non-proline residues have been assigned.

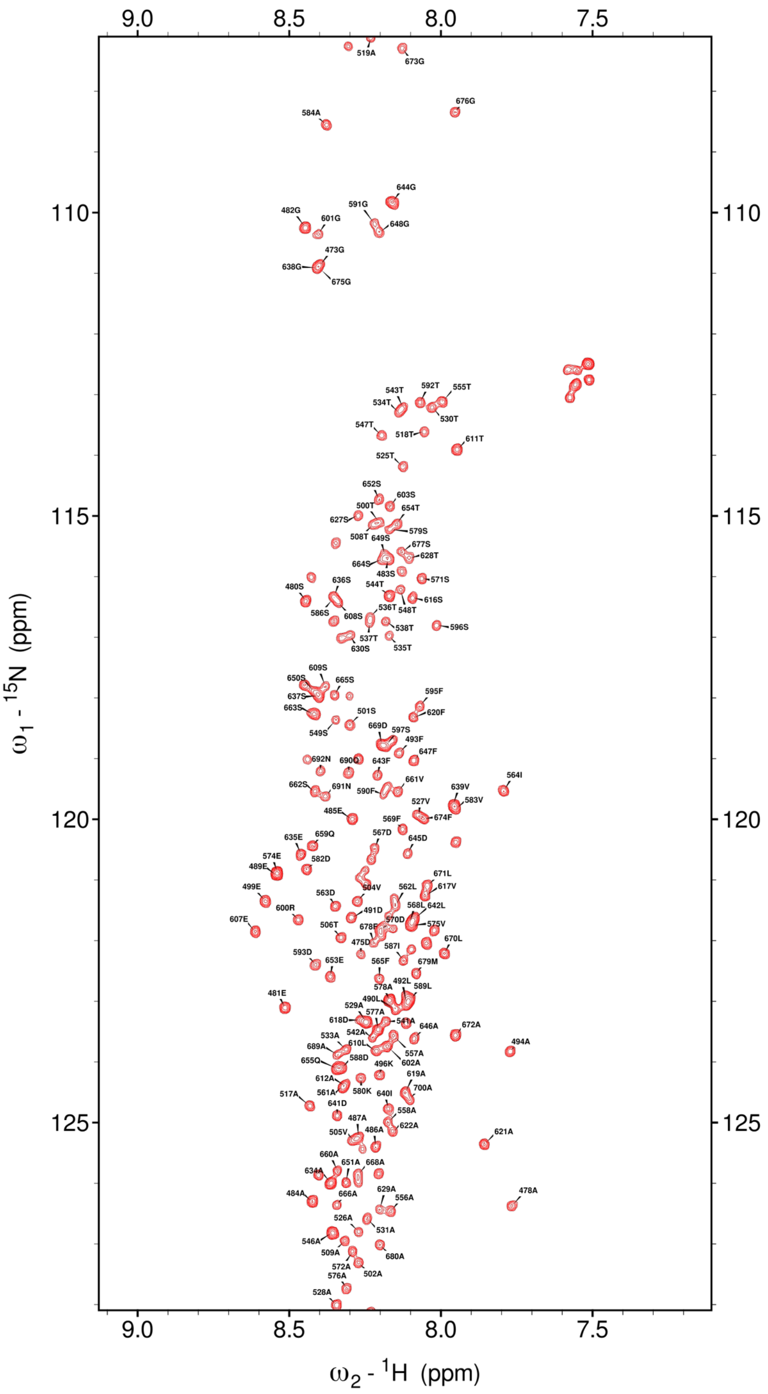

Supplementary Figure 4: Assignment of AP180$_{471\text{-}700}$. The ^{1}H-^{15}N HSQC of AP180$_{471\text{-}700}$ showing the peak assignment. Note that all numbers are shifted by +1 with respect to the protein sequence for cloning reasons. 75% of the non-proline residues have been assigned.

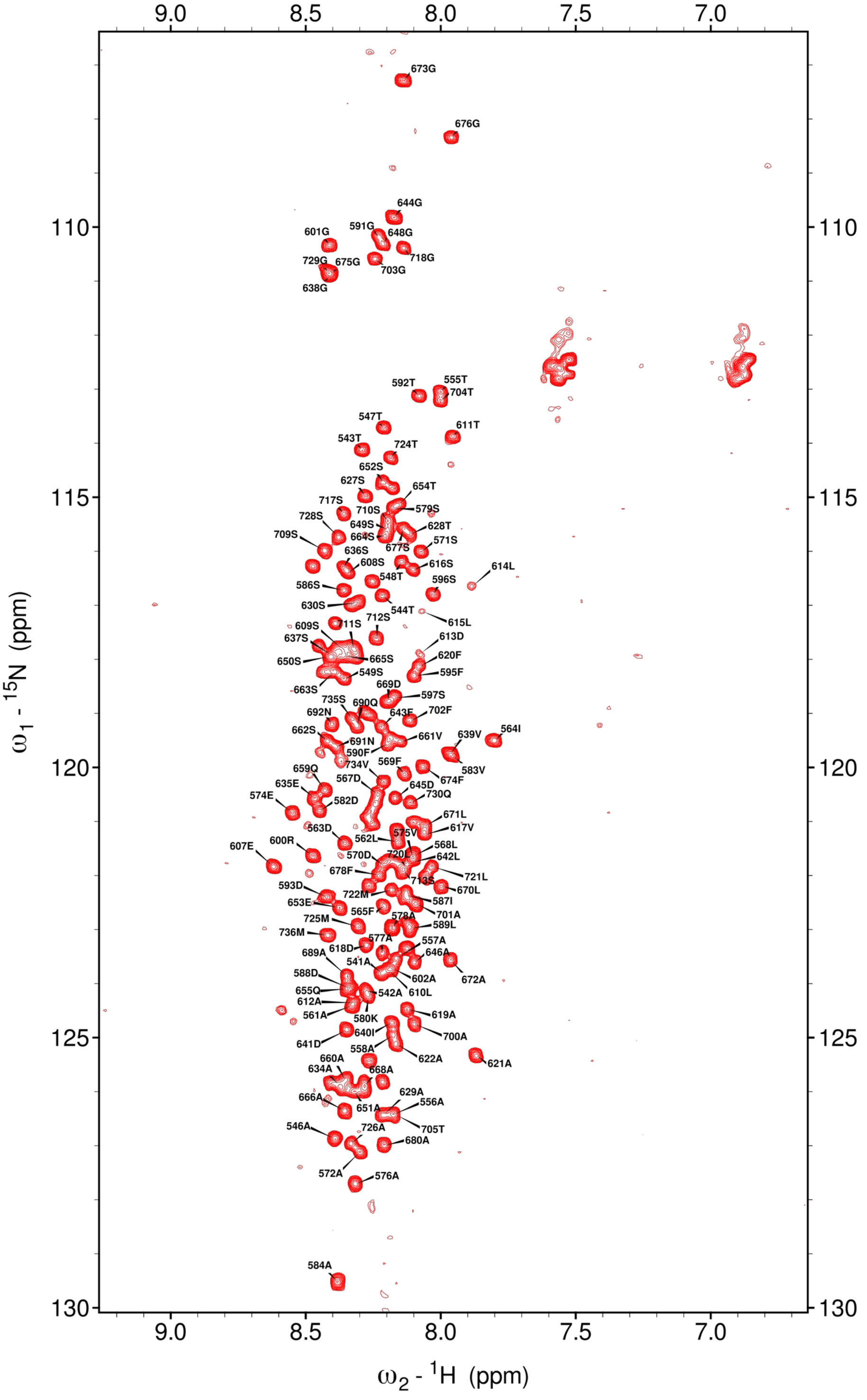

Supplementary Figure 5: Assignment of AP180$_{540\text{-}740}$. The ^{1}H-^{15}N HSQC of AP180$_{540\text{-}740}$ showing the peak assignment. Note that all numbers are shifted by +1 with respect to the protein sequence for cloning reasons. 70% of the non-proline residues have been assigned.

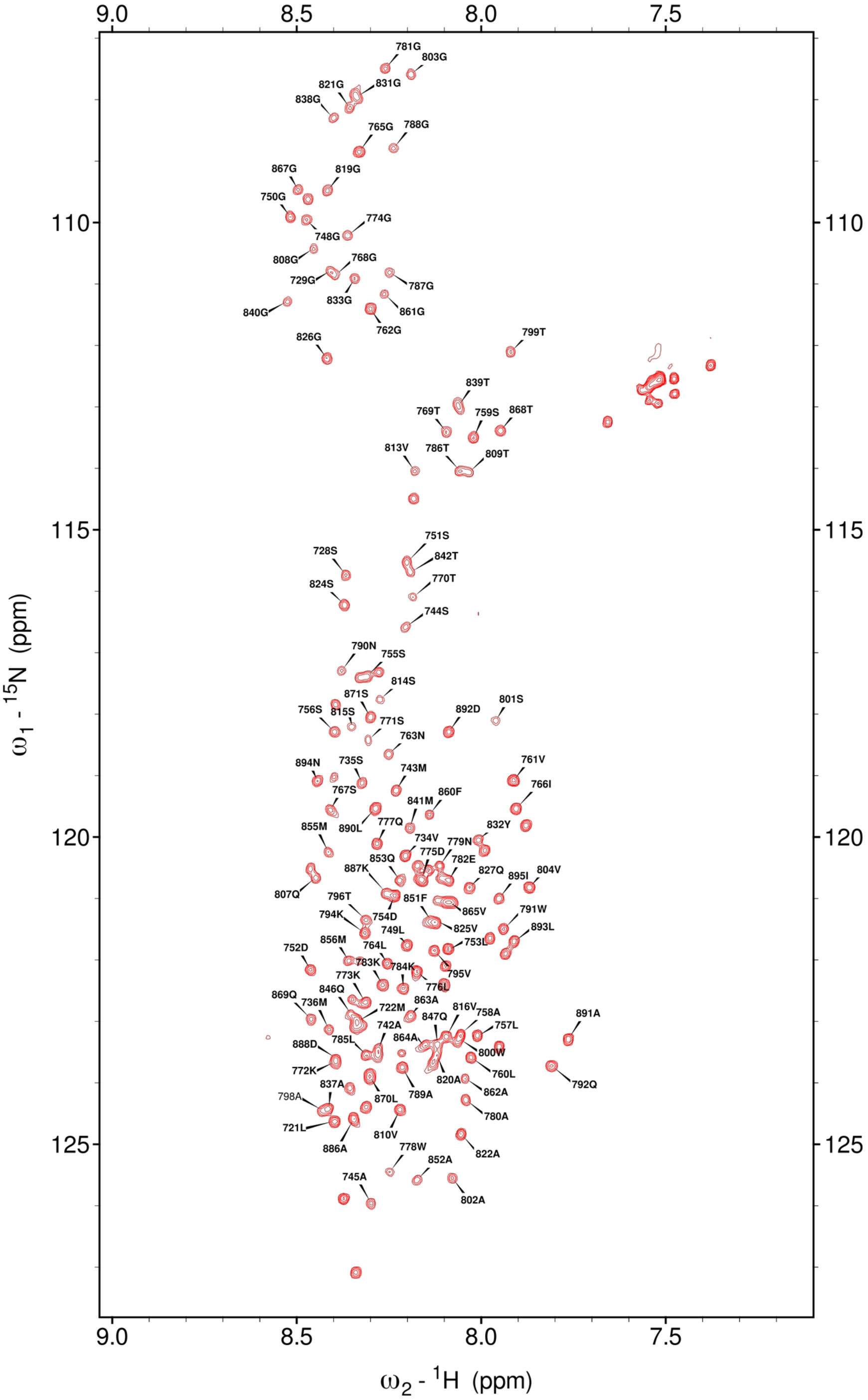

Supplementary Figure 6: Assignment of AP180$_{720\text{-}898}$. The ^{1}H-^{15}N HSQC of AP180$_{720\text{-}898}$ showing the peak assignment. Note that all numbers are shifted by +1 with respect to the protein sequence for cloning reasons. 71% of the non-proline residues have been assigned.

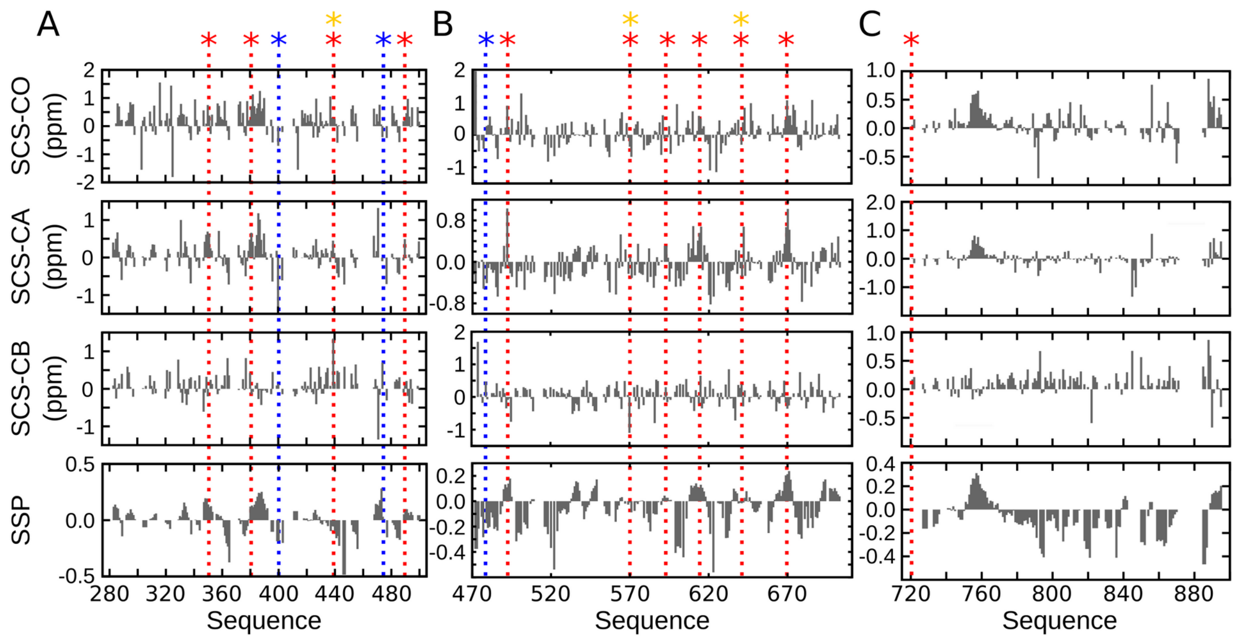

Supplementary Figure 7: Secondary structure analysis of AP180. Shown are carbon secondary chemical shifts and secondary structure propensities (SSP) calculated from Cα and Cβ chemical shifts[2]. 1: helical conformation, -1: extended conformation, values in between represent intermediate states. Shown are the data of constructs **(A)** AP180$_{281\text{-}500}$ **(B)** AP180$_{471\text{-}700}$ **(C)** AP180$_{720\text{-}898}$. Colored stars indicate putative interaction sites for clathrin (DLL/DLF) and AP2 α- and β2-appendage domains, respectively (DPF, FxDxF) (legend as in Fig. 1 and Supplementary Fig. 1).

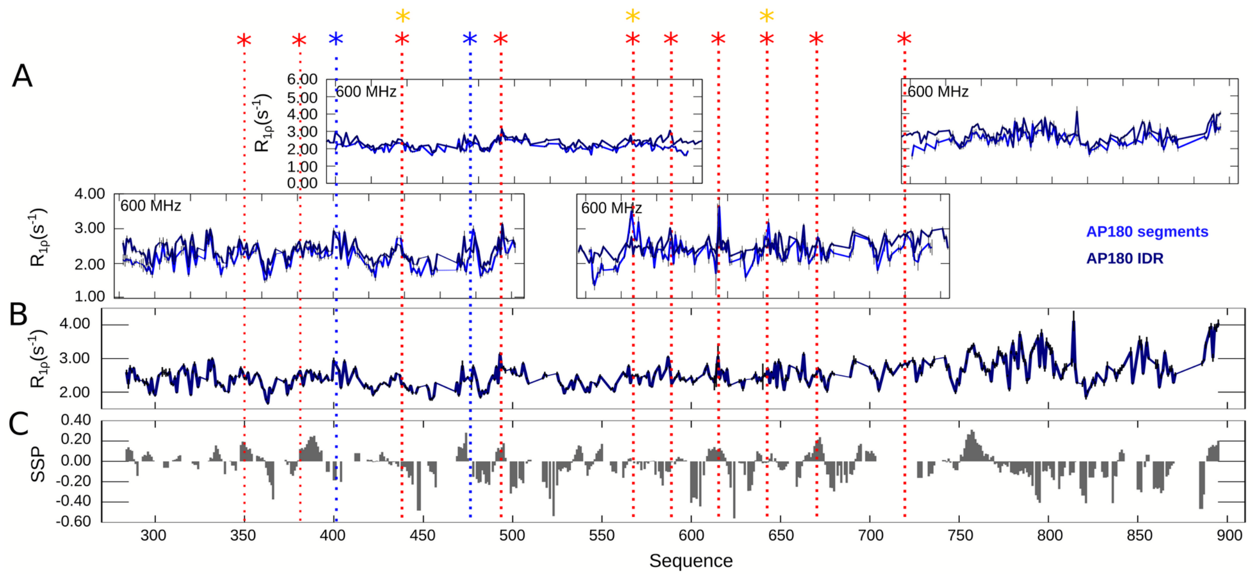

Supplementary Figure 8: Conformational dynamics of AP180 IDR. (A) ^{15}N $R_{1\rho}$ relaxation of different AP180 segments (blue) compared to the full length IDR (dark blue). Error bars are shown as black lines. **(B)** Full display of $AP180_{IDR}$ $R_{1\rho}$ (drak blue, error bars in black). **(C)** Secondary structure propensities (SSP)[2] calculated and merged from C_α and C_β chemical shifts of the different AP180 segments. Colored stars indicate putative interaction sites for clathrin (DLL/DLF) and AP2 α- and β2-appendage domains, respectively (DPF, FxDxF) (legend as in Fig. 1 and Supplementary Fig. 1).

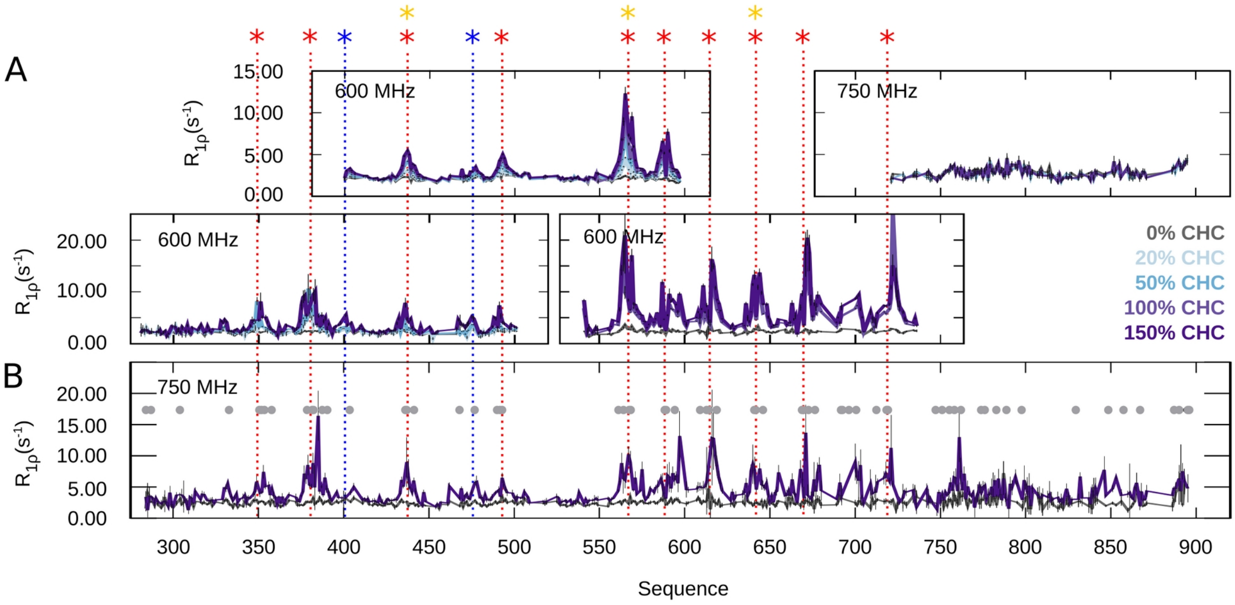

Supplementary Figure 9: Relaxation of AP180 in the absence and presence of CHC_{TD}. $R_{1\rho}$ relaxation rates are shown at an AP180 concentration of 100 µM and at increasing concentrations of CHC_{TD} (color legend shown in (A)). **(A)** Interaction of different AP180 segments with CHC_{TD}. **(B)** Interaction of $AP180_{IDR}$ with CHC_{TD}. Hydrophobic residues LFWY are shown as gray points along the sequence in (B). Field strengths (^{1}H Larmor frequencies) at which the relaxation rates were recorded are shown in the respective plots. Colored stars indicate putative interaction sites for clathrin (DLL/DLF) and AP2 α- and β2-appendage domains, respectively (DPF, FxDxF) (legend as in Fig. 1 and Supplementary Fig. 1).

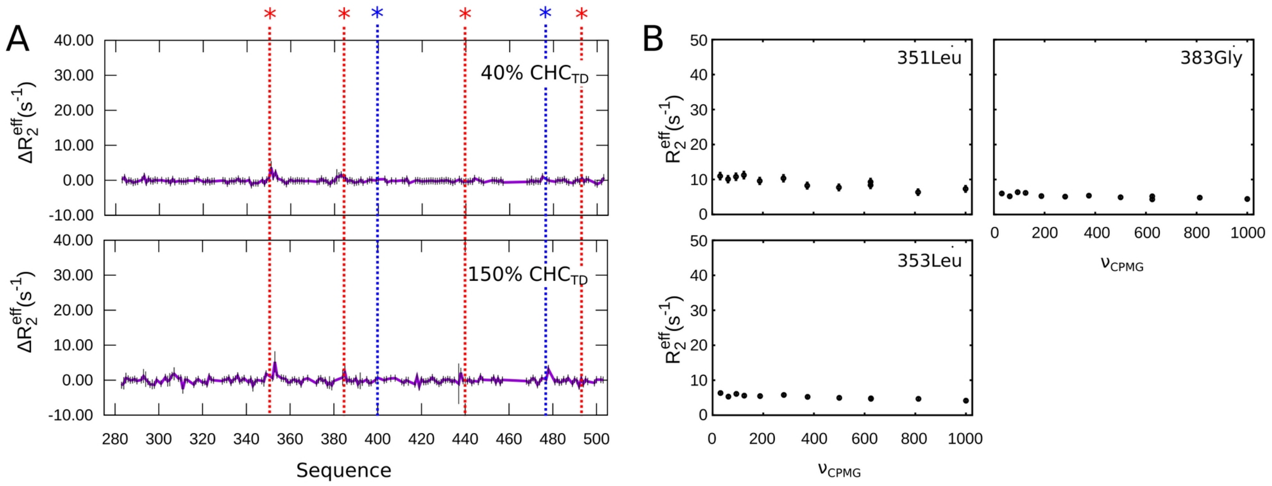

Supplementary Figure 10: CPMG relaxation dispersion of AP180$_{281\text{-}500}$ with different concentrations of CHC$_{TD}$. (A) ΔR_2^{eff} as derived from CPMG relaxation dispersion experiments of 100 µM ^{15}N AP180$_{281\text{-}500}$ with 40% and 150% CHC$_{TD}$ compared to the concentration of AP180$_{281\text{-}500}$. Plotted is the difference between R_2^{eff} at CPMG frequencies of 31.35 Hz and 1000 Hz. (B) CPMG curves of AP180$_{281\text{-}500}$ with 40% CHC$_{TD}$ for residues 351Leu, 353Leu and 383Gly, the residues showing the highest ΔR_2^{eff} in this experiment. Data were recorded at a ^{1}H Larmor frequency of 600 MHz. Errors were propagated from the errors of the CPMG experiment.

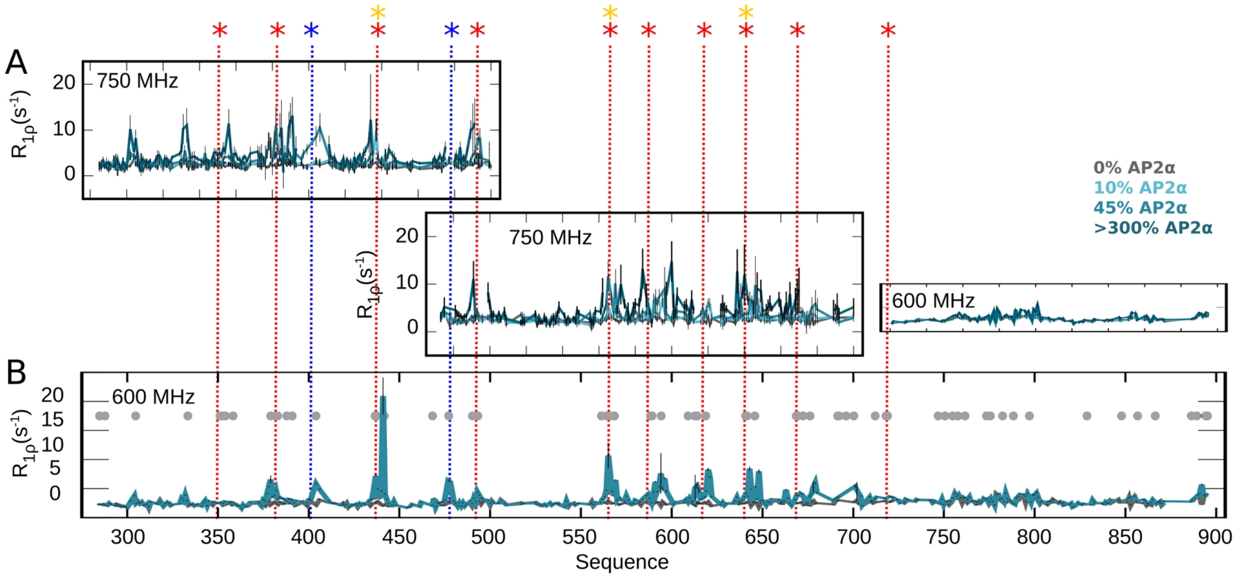

Supplementary Figure 11: Relaxation of AP180 in the absence and presence of AP2α. (A) $R_{1\rho}$ relaxation rates of AP180 segments are shown at an AP180 concentration of 50 μM and at increasing concentrations of AP2α (color legend shown in (A)). **(B)** Interaction of the full $AP180_{IDR}$ with AP2α. The concentration of $AP180_{IDR}$ in the absence and presence of 45% AP2α was 100 μM and 91 μM, respectively. Hydrophobic residues LFWY are shown as gray points along the sequence in (B). Field strengths (^{1}H Larmor frequencies) at which the relaxation rates were recorded are shown in the respective plots. Colored stars indicate putative interaction sites for clathrin (DLL/DLF) and AP2 α- and β2-appendage domains, respectively (DPF, FxDxF) (legend as in Fig. 1 and Supplementary Fig. 1).

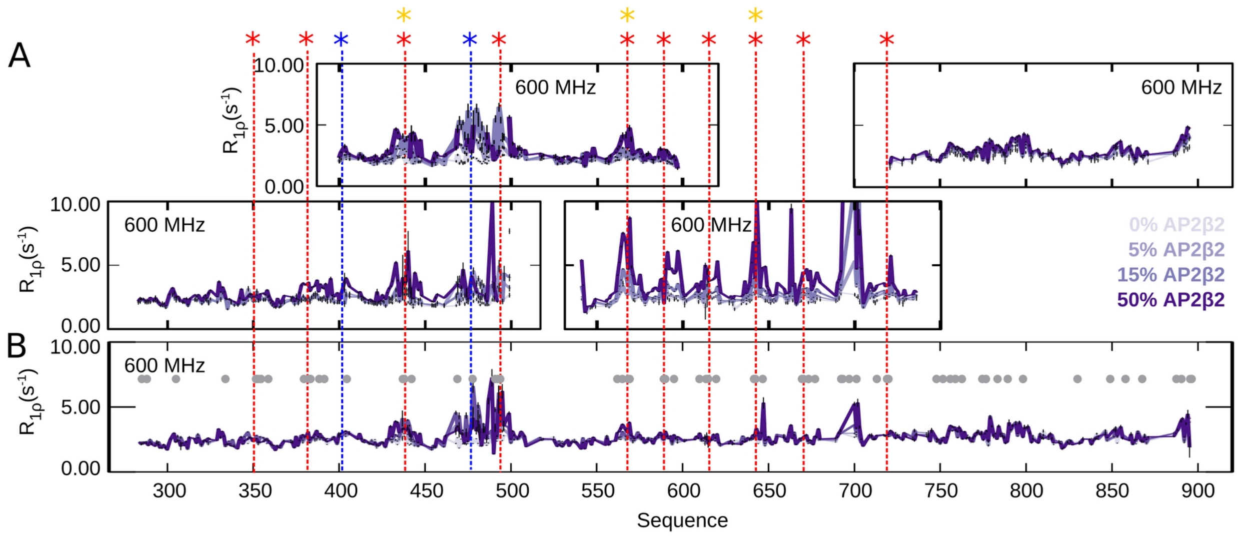

Supplementary Figure 12: Relaxation of AP180 in the absence and presence of AP2β2. (A) ^{15}N $R_{1\rho}$ relaxation rates are shown at an AP180 concentration of 100 μM and at increasing concentrations of AP2β2 (color legend shown in (A)). **(B)** Interaction of the full $AP180_{IDR}$ with AP2β2. Hydrophobic residues LFWY are shown as gray points along the sequence in (B). Field strengths (^{1}H Larmor frequencies) at which the relaxation rates were recorded are shown in the respective plots. Colored stars indicate putative interaction sites for clathrin (DLL/DLF) and AP2 α- and β2-appendage domains, respectively (DPF, FxDxF) (legend as in Fig. 1 and Supplementary Fig. 1). Note that NMR resonances around the EIM disappear already at very low concentrations of AP2β2. At high admixtures (> 15%) it was thus not possible to determine $R_{1\rho}$ rates throughout the whole interaction site, leading to apparently comparable rates around the EIM in construct $AP180_{281\text{-}500}$ or $AP180_{399\text{-}598}$ and the SLiMs in construct $AP180_{541\text{-}740}$. Domination of the EIM over SLiMs is obvious in the rates of the full $AP180_{IDR}$ (B).

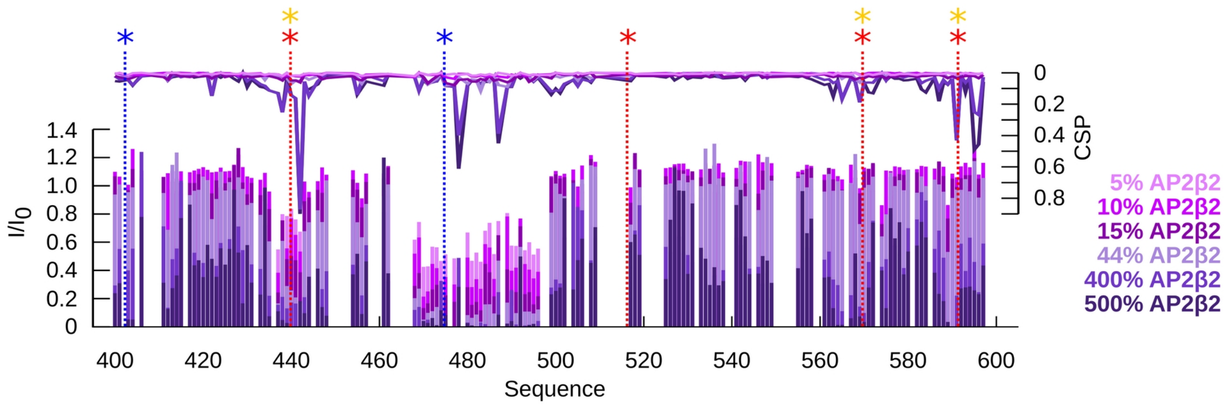

Supplementary Figure 13: Interaction of $AP180_{399-598}$ with higher concentrations of AP2β2. Top: Chemical shift perturbations of $AP180_{399-598}$ at increasing concentrations of AP2β2. Bottom: Intensity ratios (I/I_0) extracted from ^{1}H-^{15}N HSQC spectra on $AP180_{399-598}$ alone and in the presence of increasing concentrations of AP2β2. $AP180_{399-598}$ has been concentrated to 100µM in all samples. Color legend for the different AP2β2 concentrations are on the right. Colored stars indicate putative interaction sites for clathrin (DLL/DLF) and AP2 α- and β2-appendage domains, respectively (DPF, FxDxF) (legend as in Fig. 1 and Supplementary Fig. 1).

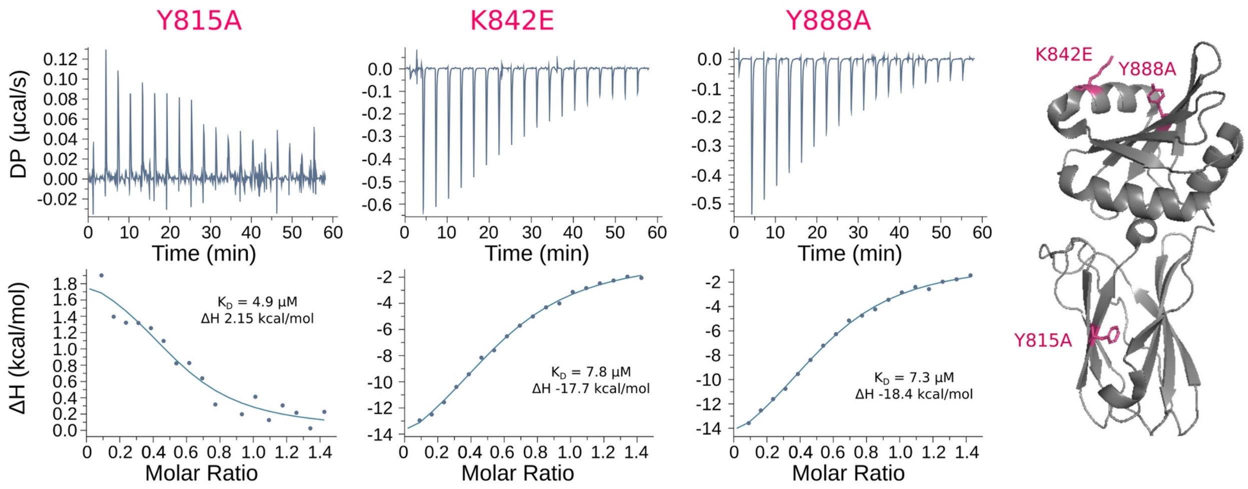

Supplementary Figure 14: ITC of AP180$_{399\text{-}598}$ with AP2β2 mutants. Differential heating powers (DP) per injection are shown on top, enthalpy versus molar ratio of the interaction partners are shown in the bottom row. The data are fitted with a 1:1 binding model resulting in the affinities indicated in the respective graphs (K_D values and binding enthalpies ΔH are shown in the respective graphs). The structure of AP2β2 with the different mutations indicated in pink is shown on the right.

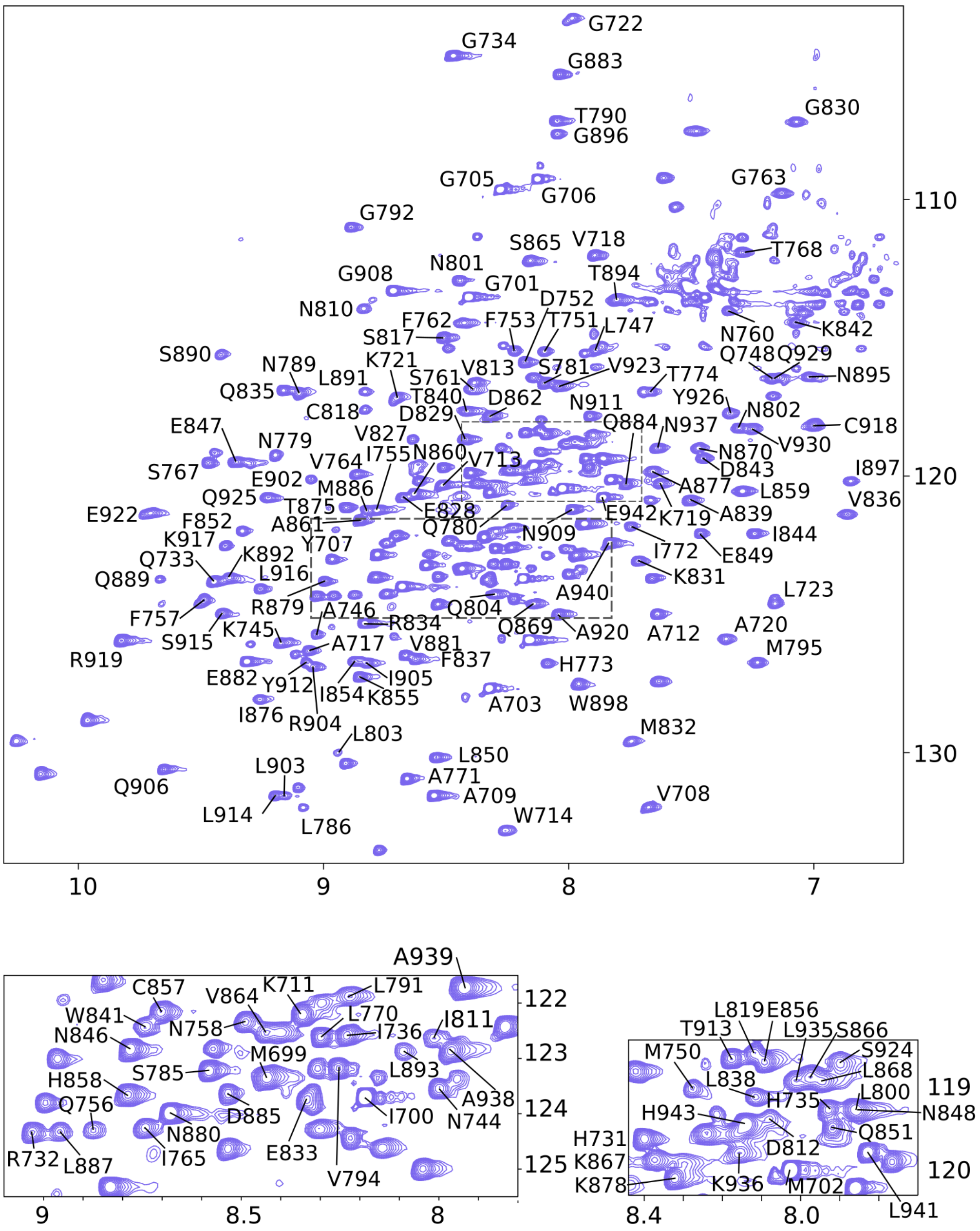

Supplementary Figure 15: Assignment of AP2β2$_{714\text{-}950}$. The ^{1}H-^{15}N TROSY of deuterated AP2β2$_{714\text{-}950}$ showing the peak assignment. Overlapped regions are shown as a zoom below the spectrum and correspond to the boxed regions within the full spectrum of AP2β2$_{714\text{-}950}$. 62% of the non-proline residues have been assigned.

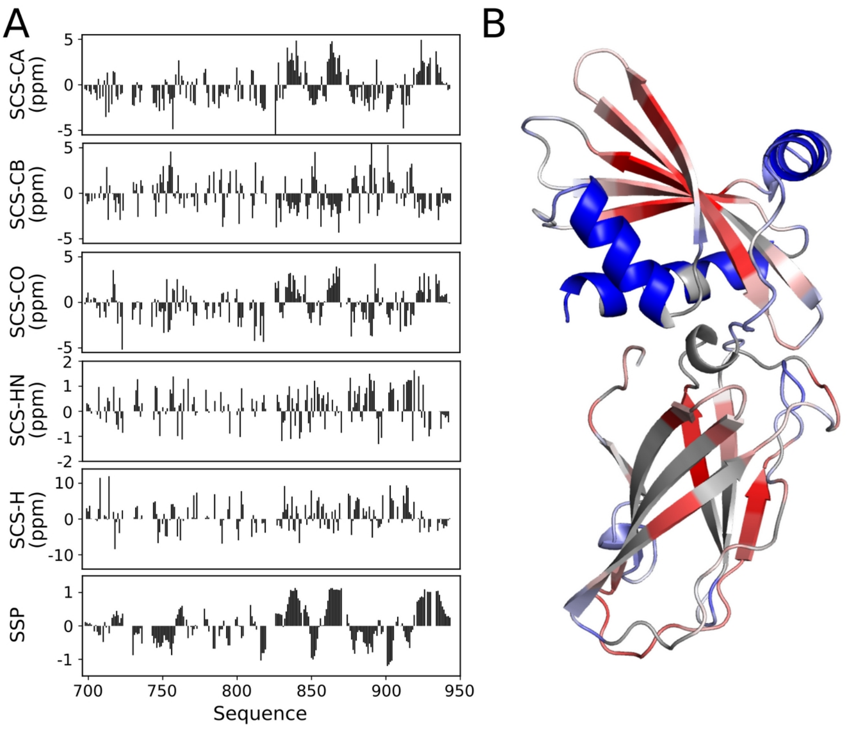

Supplementary Figure 16: Secondary structure analysis of AP2β2$_{714\text{-}950}$. (A) Secondary chemical shifts of AP2β2$_{714\text{-}950}$ and secondary structure propensities (SSP) calculated from Cα and Cβ chemical shifts[2]. 1: helical conformation, -1: extended conformation, values in between represent intermediate states. **(B)** SSPs plotted into the structure of APβ2 (PDB 1E42) with a gradient from -1 (red) to +1 (blue). Gray residues are unassigned or prolines.

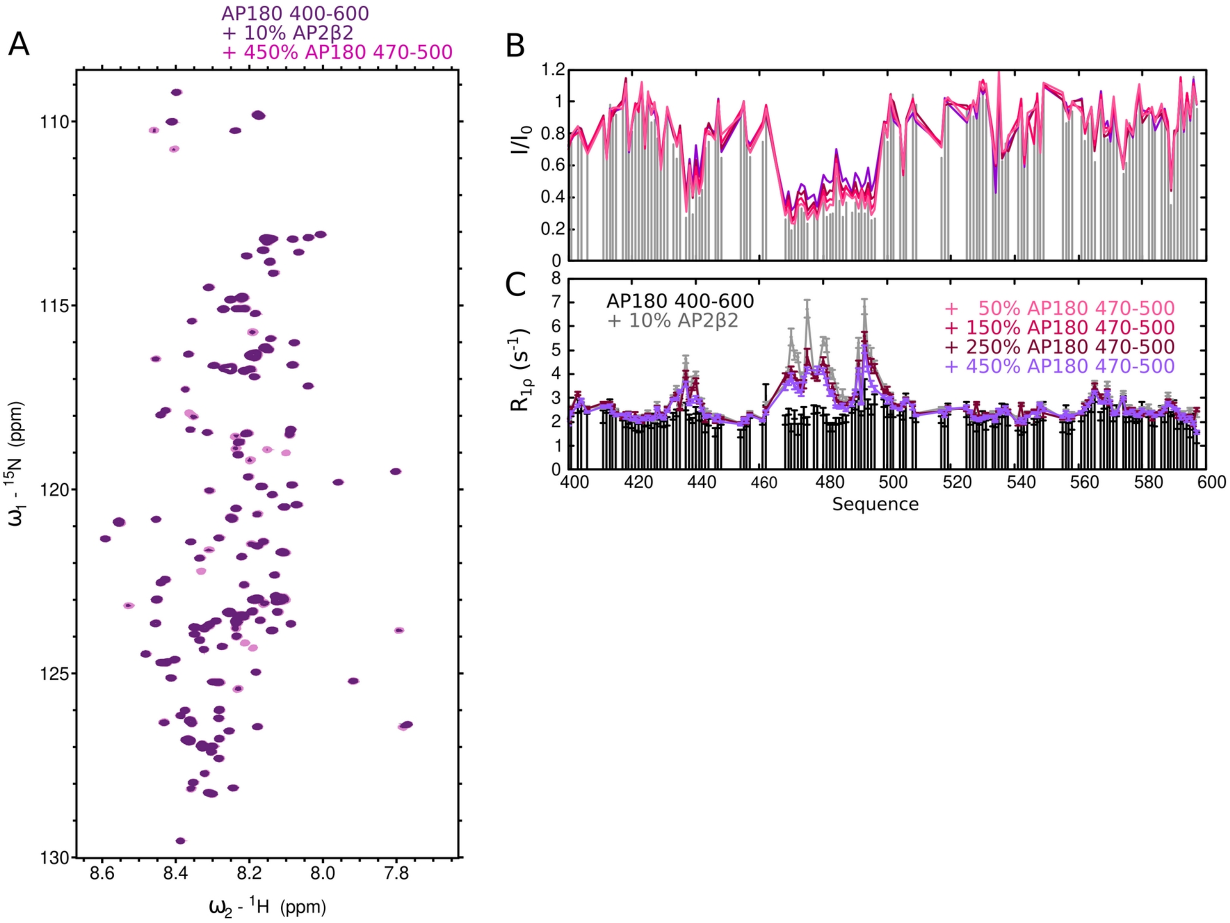

Supplementary Figure 17: Competition of $AP180_{399\text{-}598}$ from AP2β2 binding by $AP180_{470\text{-}500}$. (A) ^{1}H-^{15}N HSQC spectrum of ^{15}N $AP180_{399\text{-}598}$ at 100 µM with 10% APβ2 and additionally $AP180_{470\text{-}500}$ (color legend is indicated in the figure). **(B)** Intensity ratio (I/I_0) of $AP180_{399\text{-}598}$ peaks in the presence versus the absence of 10% AP2β2 + increasing amounts of $AP180_{470\text{-}500}$ (color legend in (C)). **(C)** ^{15}N $R_{1\rho}$ relaxation rates of $AP180_{399\text{-}598}$ in the presence of 10% AP2β2 with increasing amounts of $AP180_{470\text{-}500}$ and $AP180_{399\text{-}598}$ alone (color legend is displayed in the figure), measured at a ^{1}H Larmor frequency of 900 MHz

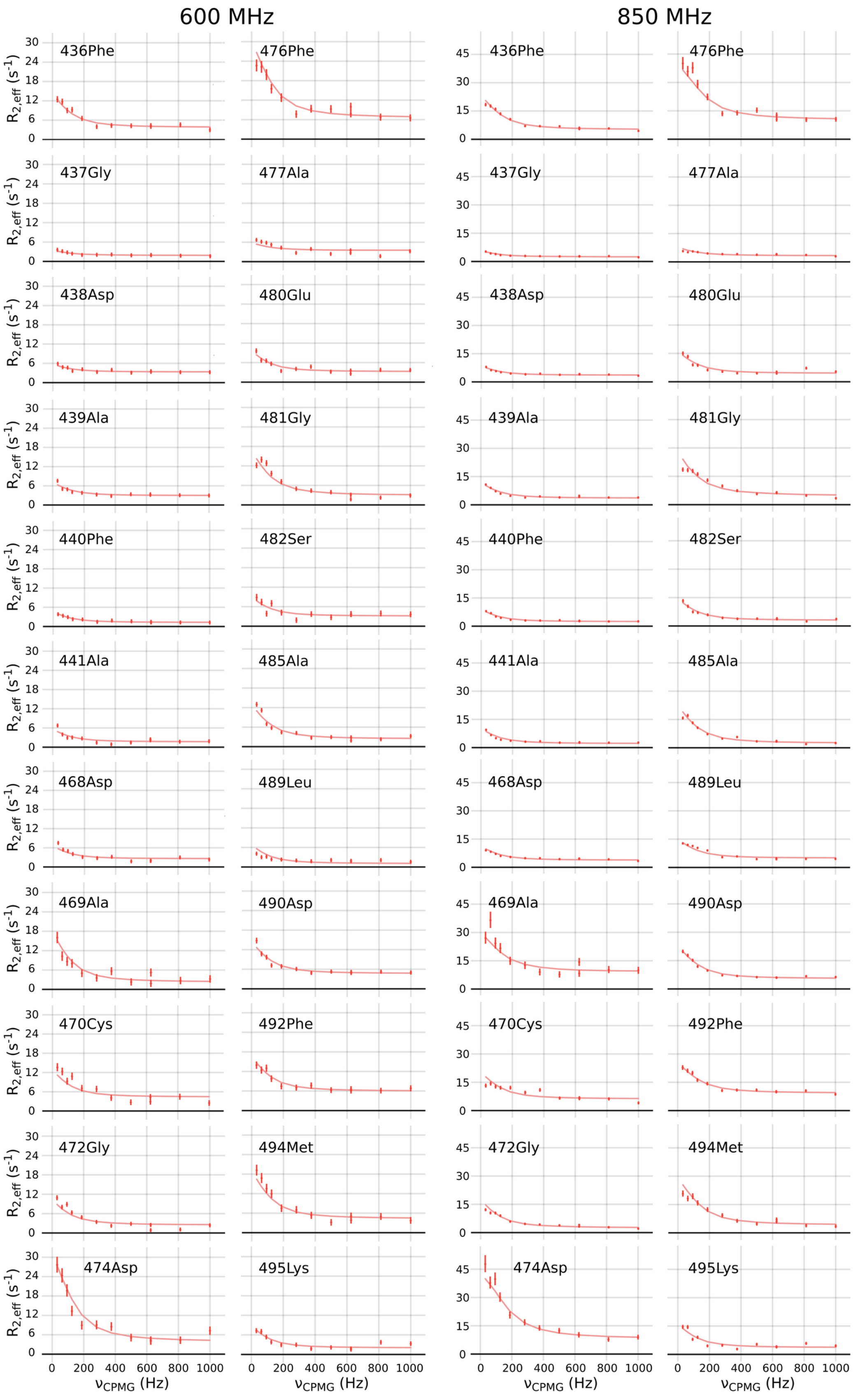

Supplementary Figure 18: CPMG relaxation dispersion of AP180$_{399\text{-}598}$ (100 µM) with 10% AP2β2. Data from all 22 sites showing relaxation dispersion at a ^{1}H frequency of 600 MHz and 850 MHz were fit using the program Chemex in a global fit, assuming a two-site exchange process

(unbound – bound). The data fitted to k_{ex} = 662±35 s^{-1} and a population of the bound state p_B = 8.9±0.97 %. Chemical shift difference obtained from the fit were in the range of 0.3 and 1 ppm.

Supplementary Tables:

Supplementary Table 1: List of clathrin binding regions. The regions are centered around the hydrophobic residue within the cluster of increased $R_{1\rho}$ rates. 10 residues left and right of that central residues were selected. The sequences were then sorted according to apparent affinities; sequences with the highest $R_{1\rho}$ rates in $AP180_{IDR}$ upon interaction with CHC_{TD} appear first.

^{371}TGGATAWGDLLGEDSLAALSS^{391}
^{660}VSSSSASADLLAGFGGSFMAP^{680}
^{584}PSIDLFGTDAFSSPPRGASPV^{604}
^{604}VPESSLTADLLSVDAFAAPSP^{624}
^{749}GSDLDSSLASLVGNLGISGTT^{769}
^{710}SSSFDPSGDLLMPTMAPSGQP^{730}
^{687}PAQNNLLQPNFEAAFGTTPST^{707}
^{554}TAAAPPALDIFGDLFDSAPEV^{574}
^{632}KAESSGVIDLFGDAFGSSASE^{652}
^{425}VTAATTEVDLFGDAFAASPGE^{445}
^{345}SSDLLDLQPDFSGAAAGAAAP^{365}
^{465}ALDACSGNDPFAPSEGSAEAA^{485}
^{481}SAEAAPELDLFAMKPPETSAP^{501}

Supplementary Table 2: Residue wise affinities in $AP180_{IDR}$ with CHC_{TD}. ^{15}N $R_{1\rho}$ relaxation rates of $AP180_{IDR}$ in the presence of various concentrations of CHC_{TD} (0%, 20%. 60%, 150%) at an AP180 concentration of 100 µM were plotted against the respective CHC concentration and fitted with a 1:1 binding model (see materials and methods for details, errors represent fitting errors). The highest $R_{1\rho}$ rates per cluster were used for this analysis, respectively. Note that this apparently highest $R_{1\rho}$ rate does not necessarily occur at a hydrophobic residue, depending on the exact binding mode of the residue and potentially influenced by spectral overlap within the large $AP180_{IDR}$. Nonetheless, the determined affinities should represent binding affinities towards individual linear motifs.

**Residue number**	**Residue type**	**Surrounding amino acids**	**Affinity ± error (µM)**
384	D	GE**D**SL	177 ± 8
670	L	DL**L**AG	294 ± 67
596	S	FS**S**PP	1507 ± 1165
615	S	LL**S**VD	719 ± 335
720	L	DL**L**MP	732 ± 320
566	D	FG**D**LF	549 ± 131
436	F	DL**F**GD	700 ± 58
699	A	FE**A**AF	1166 ± 957
639	I	GV**I**DL	846 ± 307
678	M	SF**M**AP	576 ± 10±7
378	G	AW**G**DL	650 ± 83
574	V	PE**V**AA	817 ± 133
891	D	LA**D**LN	1248 ± 864
352	L	LD**L**QP	1011 ± 214
492	F	DL**F**AM	1170 ± 215
477	A	PF**A**PS	1374 ± 308

Supplementary Table 3: Residue wise affinities in AP180 with AP2α. ^{15}N $R_{1\rho}$ relaxation rates from $AP180_{471\text{-}700}$ in the presence of various concentrations of AP2α (0%, 10%, 45%, 320%) at an AP180 concentration of 50 μM were plotted against the respective CHC concentration and fitted with a 1:1 binding model (see materials and methods for details, errors represent fitting errors). The highest $R_{1\rho}$ rates per cluster were used for this analysis, respectively. Note that this apparently highest $R_{1\rho}$ rate does not necessarily occur at a hydrophobic residue, depending on the exact binding mode of the residue and potentially influenced by spectral overlap. Nonetheless, the determined affinities should represent binding affinities towards individual linear motifs.

**Residue number**	**Residue type**	**Surrounding amino acids**	**Affinity ± error (μM)**
490	D	EL**D**LF	278 ± 83
498	E	PP**E**TS	463 ± 154
561	L	PA**L**DI	380 ± 47
571	A	DS**A**PE	320 ± 60
582	V	PD**V**AP	414 ± 27
585	S	AP**S**ID	459 ± 253
596	S	FS**S**PP	391 ± 79
599	R	PP**R**GA	234 ± 132
602	S	GA**S**PV	441 ± 156
615	S	LL**S**VD	144 ± 28
635	S	AE**S**SG	196 ± 61
639	I	GV**I**DL	257 ± 125
648	S	TT**S**AA	316 ± 58
664	S	SS**S**AS	416 ± 198
668	D	SA**D**LL	288 ± 110
669	L	AD**L**LA	273 ± 74

Supplementary Table 4: Peptides detected by mass spectrometry from AP180$_{430\text{-}500}$ cross-linking experiment. The peptides are sorted with respect to type (crosslink, monolink, looplink or regular peptide). The detected peptides are indicated; numbers in brackets denote the position of the cross-linked lysine within the detected peptide. Their molecular mass is given in daltons (Da) and peptide modifications are indicated. The number in brackets refers to the residue number within the peptide that is modified; counts start from the first peptide and go on to the second; three residues are added in addition if the modification occurs in the second peptide. The detected peptides are then associated to the protein they originate from; in brackets is the position of the cross-linked lysine with respect to the full protein sequence used in the cross-linking experiment. The amino acid sequences of both AP180$_{430\text{-}500}$, as well as APβ2$_{714\text{-}951}$ are indicated below the table.

**Crosslink**	**Peptide**	**Peptide_Mass**	**Modifications**	**Proteins**
1	AAPELDLFAMKPPE(11)-AAPELDLFAMKPPE(11)	3210.598759	Oxidation[M](10)	AP180_430-500(69)-AP180_430-500(69)
2	AAPELDLFAMKPPE(11)-LDLFAMKPPET(7)	2943.476864	Oxidation[M](10)	AP180_430-500(69)-AP180_430-500(69)
3	AAPELDLFAMKPPET(11)-LDLFAMKPPE(7)	2943.476864	Oxidation[M](24)	AP180_430-500(69)-AP180_430-500(69)
4	GSAEAAPELDLFAMKPPE(15)-LQFQIKE(6)	2931.469471	Oxidation[M](14)	AP180_430-500(69)-AP2B2(159)
5	LDLFAMKPPE(7)-LDLFAMKPPE(7)	2458.264709	null	AP180_430-500(69)-AP180_430-500(69)
6	LFAMKPPE(5)-LFAMKPPE(5)	2034.032544	Oxidation[M](4);Oxidation[M](15)	AP180_430-500(69)-AP180_430-500(69)
7	SILKNAAALE(4)-LFAMKPPE(5)	2115.140507	Oxidation[M](17)	AP2B2(240)-AP180_430-500(69)

**Monolink**	**Peptide**	**Peptide_Mass**	**Modifications**	**Proteins**
1	AAPELDLFAMKPPET(11)	1801.892749	Oxidation[M](10)	AP180_430-500(69)
2	LDLFAMKPPE(7)	1332.675507	Oxidation[M](6)	AP180_430-500(69)
3	LFAMKPPE(5)	1104.564511	Oxidation[M](4)	AP180_430-500(69)
4	LFAMKPPET(5)	1189.61727	null	AP180_430-500(69)
5	LSTGIGMAPGGYVAPKAVWLPAVKAKGLE(16)	3053.674247	Oxidation[M](7)	AP2B2(15)
6	RQVFLATWKD(9)	1419.763012	null	AP2B2(146)

**Looplink**	**Peptide**	**Peptide_Mass**	**Modifications**	**Proteins**
1	LFELSTGIGMAPGGYVAPKAVWLPAVKAKGLEISGTFTHRQGHIYME(19)(27)	5198.684345	Oxidation[M](10);Oxidation[M](46)	AP2B2(15)(23)
2	LFELSTGIGMAPGGYVAPKAVWLPAVKAKGLEISGTFTHRQGHIYME(27)(29)	5198.684345	Oxidation[M](10);Oxidation[M](46)	AP2B2(23)(25)

**Regular peptide**	**Peptide**	**Peptide_Mass**	**Modifications**	**Proteins**
1	AAPELDLFAMKPPE	1528.771523	null	AP180_430-500
2	ACSGNDPFAPSE	1194.473128	null	AP180_430-500
3	ACSGNDPFAPSEGSAE	1538.606312	null	AP180_430-500

4	AFAASPGEAPAASE	1275.585116	null	AP180_430-500
5	AFAASPGEAPAASEGATAPATPAPVAAALD	2650.29938	null	AP180_430-500
6	APAASEGATAPATPAPVAAALDACSGNDPFAPSE	3095.426095	null	AP180_430-500
7	CHLNADTVSSKLQNNNVYTIAKRNVE	2931.473989	null	AP2B2
8	DGKMERQVFLATWKDIPNE	2277.133124	null	AP2B2
9	FAIQFNKNSFGVIPSTPLAIHTPLMPNQSID	3397.761152	null	AP2B2
10	GATAPATPAPVAAALD	1393.732105	null	AP180_430-500
11	GATAPATPAPVAAALDACSGNDPFAPSE	2569.187392	null	AP180_430-500
12	GATAPATPAPVAAALDACSGNDPFAPSEGSAE	2913.320576	null	AP180_430-500
13	GKMERQVFLATWKDIPNE	2162.106185	null	AP2B2
14	GKMERQVFLATWKDIPNENE	2405.191695	null	AP2B2
15	GQDMLYQSLKLTNGIWILAE	2309.184488	Oxidation[M](4)	AP2B2
16	GSAEAAPELDLFAMKPPE	1872.904707	null	AP180_430-500
17	HHHHHH	841.371277	null	AP2B2
18	ISGTFTHRQGHIYME	1792.843448	Oxidation[M](14)	AP2B2
19	LDLFAMKPPE	1176.59687	Oxidation[M](6)	AP180_430-500
20	LFAMKPPE	948.485873	Oxidation[M](4)	AP180_430-500
21	LFAMKPPET	1033.538633	null	AP180_430-500
22	LFELSTGIGMAPGGYVAPKAVWLPAVKAKGLE	3270.795749	null	AP2B2
23	LFGDAFAASPGE	1181.54728	null	AP180_430-500
24	LFGDAFAASPGEAPAASE	1707.785982	null	AP180_430-500
25	LGGGIGGSPAVGQSFIPSSVPATFAPSPTPAVVSSGLND	3595.827701	null	AP2B2
26	LGGGIGGSPAVGQSFIPSSVPATFAPSPTPAVVSSGLNDLFE	3985.022755	null	AP2B2
27	LGPPVNVPQVSSMQMGAVD	1941.940769	Oxidation[M](15)	AP2B2
28	LGPPVNVPQVSSMQMGAVDLLGGGLD	2551.28935	null	AP2B2
29	LLGDLLNLD	985.556391	null	AP2B2
30	LLGGGLDSLLGSD	1216.641901	null	AP2B2
31	LQFQIKE	905.509052	null	AP2B2
32	LRIQPGNPNYTLSLKCRAPE	2270.207287	null	AP2B2
33	LSTGIGMAPGGYVAPKAVWLPAVKAKGLE	2881.600695	null	AP2B2
34	LSTGIGMAPGGYVAPKAVWLPAVKAKGLEISGTFTHRQGHIYME	4639.431388	null	AP2B2
35	MEQPQVIPSQGDLLGD	1726.831547	null	AP2B2
36	MLYQSLKLTNGIWILAE	1993.082603	null	AP2B2
37	MNFTNKALQHMTD	1550.708939	null	AP2B2
38	PFAPSEGSAE	991.436675	null	AP180_430-500
39	PFAPSEGSAEAAPELD	1587.717239	null	AP180_430-500
40	PLNNLQVAVKNNID	1551.848856	null	AP2B2

41	QPQVIPSQGD	1068.531965	null	AP2B2
42	QPQVIPSQGDLLGD	1466.74848	null	AP2B2
43	RQVFLATWKD	1263.684375	null	AP2B2
44	RQVFLATWKDIPNE	1716.906702	null	AP2B2
45	RQVFLATWKDIPNENE	1959.992212	null	AP2B2
46	SILKNAAALE	1029.593836	null	AP2B2
47	SILKNAAALEHHHHHH	1851.947271	null	AP2B2
48	SLLGSDLGGGIGGSPAVGQSFIPSSVPATFAPSPTPAVVSSGLND	4168.108265	null	AP2B2
49	SLLGSDLGGGIGGSPAVGQSFIPSSVPATFAPSPTPAVVSSGLNDLFE	4557.303319	null	AP2B2
50	TVSSKLQNNNVYTIAKRNVE	2278.214874	null	AP2B2
51	VDLFGDAFAASPGEAPAASE	1921.881329	null	AP180_430-500
52	VSLPLNTLGPVMKMEPLNNLQVAVKNNID	3161.705949	null	AP2B2
53	VSQYIYQVYD	1277.604791	null	AP2B2
54	VSQYIYQVYDSILKNAAALE	2288.180785	null	AP2B2

Amino acid sequence of $AP180_{430\text{-}500}$:

GHMTTEVDLFGDAFAASPGEAPAASEGATAPATPAPVAAALDACSGNDPFAPSEGSAEAAPELDLFAMKPPETSLEHHHHHH

Amino acid sequence of $AP\beta2_{714\text{-}951}$:

GHMIGMAPGGYVAPKAVWLPAVKAKGLEISGTFTHRQGHIYMEMNFTNKALQHMTDFAIQFNKNSFGVIPSTPLAIHTPLMPNQSIDVSLPLNTLGPVMKMEPLNNLQVA

VKNNIDVFYFSCLIPLNVLFVEDGKMERQVFLATWKDIPNENELQFQIKECHLNADTVSSKLQNNNVYTIAKRNVEGQDMLYQSLKLTNGIWILAELRIQPGNPNYTLSL

KCRAPEVSQYIYQVYDSILKNAAALEHHHHHH

Supplementary References:

1. Erdős, G. & Dosztányi, Z. Analyzing Protein Disorder with IUPred2A. *Current Protocols in Bioinformatics* **70**, e99 (2020).
2. Marsh, J. A., Singh, V. K., Jia, Z. & Forman-Kay, J. D. Sensitivity of secondary structure propensities to sequence differences between α- and γ-synuclein: Implications for fibrillation. *Protein Sci* **15**, 2795–2804 (2006).
