# Supplementary material for: An extended interaction site determines binding between AP180 and AP2 in clathrin mediated endocytosis
Source: Nat Commun. 2024 Jul 13;15:5884. doi: 10.1038/s41467-024-50212-4 (PMC11246429; doi:10.1038/s41467-024-50212-4)
Supplement: Supplementary file 3 — Reporting Summary [file 41467_2024_50212_MOESM3_ESM.pdf]

nature portfolio

Corresponding author(s): Sigrid Milles

Last updated by author(s): Jun 28, 2024

Reporting Summary

Nature Portfolio wishes to improve the reproducibility of the work that we publish. This form provides structure for consistency and transparency in reporting. For further information on Nature Portfolio policies, see our Editorial Policies and the Editorial Policy Checklist.

Statistics

For all statistical analyses, confirm that the following items are present in the figure legend, table legend, main text, or Methods section.

n/a	Confirmed	
☐	☒	The exact sample size (n) for each experimental group/condition, given as a discrete number and unit of measurement
☐	☒	A statement on whether measurements were taken from distinct samples or whether the same sample was measured repeatedly
☒	☐	The statistical test(s) used AND whether they are one- or two-sided<br>*Only common tests should be described solely by name; describe more complex techniques in the Methods section.*
☒	☐	A description of all covariates tested
☒	☐	A description of any assumptions or corrections, such as tests of normality and adjustment for multiple comparisons
☐	☒	A full description of the statistical parameters including central tendency (e.g. means) or other basic estimates (e.g. regression coefficient) AND variation (e.g. standard deviation) or associated estimates of uncertainty (e.g. confidence intervals)
☒	☐	For null hypothesis testing, the test statistic (e.g. F, t, r) with confidence intervals, effect sizes, degrees of freedom and P value noted<br>*Give P values as exact values whenever suitable.*
☒	☐	For Bayesian analysis, information on the choice of priors and Markov chain Monte Carlo settings
☒	☐	For hierarchical and complex designs, identification of the appropriate level for tests and full reporting of outcomes
☒	☐	Estimates of effect sizes (e.g. Cohen's d, Pearson's r), indicating how they were calculated

Our web collection on statistics for biologists contains articles on many of the points above.

Software and code

Policy information about availability of computer code

Data collection	NMR Data acquisition: TopSpin 3.5, MS Data acquisition: Thermo Scientific Xcalibur 4.5.474.0 on Orbitrap Exploris 480
Data analysis	NMR chemical exchange data: Chemex 0.6.1 (https://github.com/gbouvignies/ChemEx)<br>Analysis of NMR spectra: NMRPipe 10.9, NMRFAM Sparky, CcpNmrAnalysis v 3.1.1, qMDD NMR 2.7<br>NMR backbone assignment: MARS 1.0<br>ITC data: Origin version 7.0 from MicroCal<br>Mass spectrometry data: pLink 2.3.11

For manuscripts utilizing custom algorithms or software that are central to the research but not yet described in published literature, software must be made available to editors and reviewers. We strongly encourage code deposition in a community repository (e.g. GitHub). See the Nature Portfolio guidelines for submitting code & software for further information.

Data

Policy information about availability of data

All manuscripts must include a data availability statement. This statement should provide the following information, where applicable:

- Accession codes, unique identifiers, or web links for publicly available datasets
- A description of any restrictions on data availability
- For clinical datasets or third party data, please ensure that the statement adheres to our policy

Data supporting the findings of this paper are available from the corresponding author upon request and can be found in the Source Data file. Cross-link MS data generated in this study have been deposited on proteomeXchange under the accession code PXD049300 [https://www.ebi.ac.uk/pride/archive/projects/PXD049300/private] . NMR assignments generated in this study have been deposited in the Biological Magnetic Resonance Bank under accession numbers
52320 [https://bmrb.io/data_library/summary/index.php?bmrbId=52320] (AP2β2),
52322 [https://bmrb.io/data_library/summary/index.php?bmrbId=52322] (AP180281-500),
52323 [https://bmrb.io/data_library/summary/index.php?bmrbId=52323] (AP180399-598),
52324 [https://bmrb.io/data_library/summary/index.php?bmrbId=52324] (AP180471-700),
52325 [https://bmrb.io/data_library/summary/index.php?bmrbId=52325] (AP180540-740), and
52326 [https://bmrb.io/data_library/summary/index.php?bmrbId=52326] (AP180720-898).
The PDB entry of AP2β2 used in this work is: 1E42 [https://www.rcsb.org/structure/1E42] (Beta2-adaptin appendage domain, from clathrin adaptor AP2).

Research involving human participants, their data, or biological material

Policy information about studies with human participants or human data. See also policy information about sex, gender (identity/presentation), and sexual orientation and race, ethnicity and racism.

Reporting on sex and gender	No research involving human participants, their data or their biological material has been conducted.
Reporting on race, ethnicity, or other socially relevant groupings	No research involving human participants, their data or their biological material has been conducted.
Population characteristics	No research involving human participants, their data or their biological material has been conducted.
Recruitment	No research involving human participants, their data or their biological material has been conducted.
Ethics oversight	No research involving human participants, their data or their biological material has been conducted.

Note that full information on the approval of the study protocol must also be provided in the manuscript.

Field-specific reporting

Please select the one below that is the best fit for your research. If you are not sure, read the appropriate sections before making your selection.

☒ Life sciences ☐ Behavioural & social sciences ☐ Ecological, evolutionary & environmental sciences

For a reference copy of the document with all sections, see nature.com/documents/nr-reporting-summary-flat.pdf

Life sciences study design

All studies must disclose on these points even when the disclosure is negative.

Sample size	Statistical methods were not used to determine sample size.
Data exclusions	Data were not excluded.
Replication	ITC experiments were repeated three times/twice. All attempts to repeat NMR data were successful.
Randomization	Samples were not randomized.
Blinding	Blinding was not necessary for this study.

Reporting for specific materials, systems and methods

We require information from authors about some types of materials, experimental systems and methods used in many studies. Here, indicate whether each material, system or method listed is relevant to your study. If you are not sure if a list item applies to your research, read the appropriate section before selecting a response.

Materials & experimental systems

n/a	Involved in the study
☒	☐ Antibodies
☒	☐ Eukaryotic cell lines
☒	☐ Palaeontology and archaeology
☒	☐ Animals and other organisms
☒	☐ Clinical data
☒	☐ Dual use research of concern
☒	☐ Plants

Methods

n/a	Involved in the study
☒	☐ ChIP-seq
☒	☐ Flow cytometry
☒	☐ MRI-based neuroimaging

Plants

Seed stocks	No plants were used.
Novel plant genotypes	No plants were used.
Authentication	No plants were used.
